# Carbon-ion radiotherapy induces ferroptosis and M1 macrophage polarization to inhibit the development of gastric cancer by downregulating DHODH

**DOI:** 10.3389/fmed.2025.1592116

**Published:** 2025-08-22

**Authors:** Yue Wang, Hongyi Cai

**Affiliations:** First Clinical Medical School, Gansu University of Chinese Medicine, Lanzhou, Gansu Province, China

**Keywords:** gastric cancer, CIRT, DHODH, macrophages, ferroptosis

## Abstract

**Background:**

Carbon-ion radiotherapy (CIRT) is an advanced form of high linear energy transfer (LET) radiotherapy that has demonstrated superior biological effectiveness compared to conventional photon therapy in the treatment of various malignancies; however, its role in gastric cancer remains unclear. Dihydroorotate dehydrogenase (DHODH), a key enzyme implicated in cancer progression, has been linked to tumor radiosensitivity. This study aims to investigate whether CIRT inhibits gastric cancer progression via the regulation of DHODH.

**Methods:**

Human gastric cancer cell lines (HGC27, AGS) were treated with CIRT (0 Gy, 2 Gy, and 4 Gy). Cell viability, migration, and invasion were assessed with MTT and Transwell assays. Expression of ferroptosis-related markers and DHODH was evaluated using Western blotting and quantitative reverse transcription polymerase chain reaction (qRT-PCR). Macrophage polarization was assessed by flow cytometry after exposure to tumor-conditioned medium (CM). BALB/c nude mice were subcutaneously injected with AGS cells and randomly assigned to the control, CIRT, and DHODH+CIRT groups.

**Results:**

*In vitro*, CIRT suppressed DHODH expression and enhanced intracellular iron and reactive oxygen species (ROS) accumulation, promoting ferroptosis in gastric cancer cells. CM from irradiated cells increased the CD86^+^CD206^−^ macrophage population and upregulated M1-associated cytokines. *In vivo*, CIRT significantly reduced tumor growth in xenograft models, and this effect was attenuated by DHODH overexpression. Tumor tissues from the CIRT group exhibited increased ferroptosis marker ACSL4 and reduced GPX4 expression, consistent with *in vitro* findings.

**Conclusion:**

These findings suggest that CIRT promotes ferroptosis and drives M1-like macrophage polarization through DHODH suppression. Targeting DHODH may enhance the therapeutic efficacy of CIRT in gastric cancer.

## 1 Introduction

Gastric cancer remains one of the most prevalent malignancies globally, ranking as the fifth most commonly diagnosed cancer and the fourth leading cause of cancer-related mortality worldwide (1, 2). The main treatment for local gastric cancer involves gastrectomy combined with comprehensive lymphadenectomy and adjuvant chemotherapy (3). However, due to the high likelihood of advanced disease at the time of diagnosis, many patients present with metastases, rendering complete surgical resection unfeasible (4, 5). Therefore, the development of more effective and broadly applicable therapeutic strategies is urgently needed.

Radiation therapy (RT) plays an indispensable role in cancer treatment, particularly for tumors that are unresectable or exhibit residual disease following surgery (6). However, for cancer patients with metastases to critical organs such as the brainstem and spinal cord, traditional photon radiotherapy often fails to deliver a sufficiently high dose to the tumor without causing significant acute or chronic toxicity (7, 8). Carbon-ion radiotherapy (CIRT) is an advanced form of radiotherapy characterized by high linear energy transfer (LET) and the presence of a Bragg peak, which allows for precise dose delivery to the tumor with minimal damage to surrounding normal tissues. Compared to photon-based radiotherapy, CIRT offers distinct physical and biological benefits (9). Primarily, the fundamental characteristics of carbon ion beams result in a more favorable dose distribution profile (10), allowing for equal or greater doses to be delivered to tumor tissues while minimizing radiation exposure to surrounding healthy tissues (11). Moreover, as carbon ion beams exhibit high LET, they demonstrate a significantly higher relative biological effectiveness (RBE) than photon beams (12). This enhanced RBE implies that lower radiation doses are sufficient to produce equivalent biological effects (13, 14). Additionally, when compared with proton therapy, which also exploits the Bragg peak but has a lower LET, CIRT exhibits a higher RBE (15). Advanced gastric tumors frequently develop hypoxic regions, which significantly reduce radiation-induced DNA damage and contribute to intrinsic treatment resistance (16). Even with advanced photon-based modalities such as volumetric-modulated arc therapy (VMAT), achieving optimal local control remains challenging due to the anatomical complexity and large irradiation fields required in gastric cancer. Consequently, disease control often relies heavily on surgical resection or systemic chemotherapy (17). In contrast, CIRT, due to its high LET and increased RBE, has shown promise in managing radioresistant and deep-seated tumors. Its physical and biological precision makes it a promising approach for gastrointestinal tumors that are difficult to manage with conventional radiotherapy (18). However, direct clinical evidence supporting the efficacy of CIRT in gastric cancer remains limited and largely unpublished to date, highlighting the need for further preclinical and mechanistic investigations.

Ferroptosis, a distinct form of programmed cell death first identified in 2012, is fundamentally driven by the interaction between reactive oxygen species (ROS) and polyunsaturated fatty acids (PUFAs) within membrane phospholipids, leading to excessive lipid peroxide (LPO) accumulation that compromises membrane integrity and ultimately results in cell death (19). Unlike necrosis, apoptosis, or autophagy, ferroptosis exhibits unique morphological features, including mitochondrial shrinkage, rupture of the outer membrane, and disappearance of cristae (20). This process has been closely linked to cancer development and progression. Furthermore, He et al. demonstrated that β-elemene effectively regulates radioresistance in gastric cancer by targeting the GPX4 pathway and inducing ferroptosis (21). Wang et al. (22) demonstrated that MON@PG nanoparticles represent a promising radiosensitizing strategy for gastric cancer by disrupting cellular redox homeostasis and promoting ferroptosis. Collectively, these findings underscore the considerable potential of targeting ferroptosis as a strategy for eradicating gastric cancer. Among the key regulators of ferroptosis, dihydroorotate dehydrogenase (DHODH) plays a critical role in mitochondrial lipid peroxidation defense (23). DHODH, an enzyme essential for the *de novo* synthesis of pyrimidine, participates in the pathological processes of various diseases (24). In gastric cancer, the upregulation of DHODH has been shown to contribute to chemoresistance by enhancing glycolysis (25). DHODH is consistently overexpressed in gastric cancer tissues, and its elevated expression attenuates the tumor-suppressive effects of polymerase theta on the malignant phenotype of gastric cancer cells, suggesting that DHODH may play a promotive role in tumor progression (26, 27). Moreover, emerging evidence indicates that DHODH inhibition enhances the radiosensitivity of cancer cells, further highlighting its potential as a therapeutic target (28). Furthermore, downregulation of DHODH attenuates stemness and ferroptosis resistance in gastric cancer cells (27). Targeting DHODH has been shown to enhance ferroptosis under oxidative stress, making it a potential radiosensitizing target in high oxidative stress environments such as CIRT.

This study aims to elucidate a specific mechanistic pathway by which CIRT modulates ferroptosis and macrophage polarization via DHODH inhibition in gastric cancer. These findings may provide preliminary biological insight into the therapeutic mechanisms of CIRT and highlight potential targets for enhancing its efficacy in resistant tumors.

## 2 Materials and methods

### 2.1 Cell culture

Human gastric cancer cell lines, including HGC27 (CL-0107, Procell, Wuhan, China) and AGS (CRL-173, ATCC, Rockville, MD, USA), were cultured using RPMI-1640 medium (PM150110, Procell), containing with 10% fetal bovine serum (FBS; 164210, Procell) and 1% penicillin–streptomycin solution (PSS; PB180120, Procell). Human monocytes THP-1 (CL-0233, Procell) were cultured in RPMI-1640 medium supplemented with 10% FBS, 0.05 mM β-mercaptoethanol (M8210, Solarbio, Beijing, China), and 1% PSS. All cells were incubated in a BB15 CO_2_ incubator (51023126, Thermo, Waltham, Massachusetts, USA) at 5% CO_2_, 37 °C, and saturated humidity. Following transfection and CIRT, AGS cells were treated with 40 μM erastin (IE0310, Solarbio) for 24 h to induce ferroptosis (29).

### 2.2 Cell transfection

The plasmids of overexpression DHODH (F133913) and the plasmids of negative control (NC; VT8001) were synthesized from YouBio (Changsha, China). For overexpression, AGS and HGC27 cells were transfected with 2.5 μg DHODH overexpression plasmid using Lipofectamine 3,000 (Invitrogen, USA) according to the manufacturer’s instructions. Briefly, 1 × 10^5^ cells were seeded in six-well plates and transfected at 60–70% confluence. Each well received 2.5 μg plasmid DNA and 5 μL of Lipofectamine 3,000 diluted in Opti-MEM medium (Thermo Fisher). The medium was replaced with complete RPMI-1640 after 6 h. Cells were collected for further experiments 24–48 h post-transfection. NC groups received an empty vector (pcDNA3.1). Transfection efficiency was verified by qRT-PCR and Western blot.

### 2.3 CIRT treatment

To better illustrate the physical characteristics of the carbon-ion irradiation used in our *in vitro* setup, we have included a schematic representation of the dose and LET distribution across the irradiation field (Supplementary Figure S1). The cell monolayer was positioned at the center of the spread-out Bragg peak (SOBP) region, where both the physical dose and LET values are relatively stable and biologically effective. While it is technically challenging to achieve a pristine LET in a monolayer system, care was taken to avoid the distal fall-off region and ensure consistency across the irradiated area. The monoenergetic beam was calibrated using a tissue-equivalent ionization chamber and Gafchromic film to validate both dose and LET characteristics at the cell layer depth. As previously described (30), HGC27 and AGS cells cultured in T25 flasks were irradiated using an intensity-modulated raster scanning system (IONTRIS; Siemens, Munich, Germany) at an energy of 333.82  MeV/u. All irradiation doses were administered as physical doses. The LET for carbon-ion radiation was set at 29.1351  keV/μm. The beamline was oriented horizontally, and the Bragg peak of the carbon-ion beam was precisely aligned with the solid surface to which the cells were adherent. Cells were irradiated with physical doses of 0 Gy, 2 Gy, or 4 Gy every 24 h and harvested 24 h after each exposure for subsequent analyses, including RNA extraction, Western blotting, and flow cytometry.

### 2.4 Macrophage polarization induction

THP-1 cells were first polarized to M0 macrophages by culturing in RPMI-1640 complete medium containing 150 ng/mL of phorbol ester (PMA; P6741, Solarbio) for 48 h (31). Culture supernatants (conditioned medium, CM) were collected from HGC27 and AGS cells 24 h after treatment with transfection, CIRT, or erastin and labeled as HGC27-CM or AGS-CM accordingly. These CM samples were then used to stimulate M0 macrophages for polarization toward an M1 phenotype.

### 2.5 MTT assays

For MTT assays, HGC27 and AGS cells were first irradiated once with physical doses of 0 Gy, 2 Gy, or 4 Gy using the carbon-ion beam (as described above) in T25 flasks. After irradiation, cells were harvested and reseeded into 96-well plates at a density of 3 × 10^3^ cells per well. Cell viability was then assessed via MTT assay 24 h after reseeding. This workflow ensured uniform irradiation conditions with validated LET and dose accuracy. The cultured medium of HGC27 and AGS cells was replaced with 90 μL of FBS-free RPMI-1640 medium and 10 μL of 5 mg/mL of MTT solution (C0009S, Beyotime). After the cells were incubated for 4 h, the culture medium was replaced with 100 μL of formazan-dissolved solution for further incubation until all the formazan crystals were dissolved. Finally, the optical density (OD) value of the HGC27 and AGS cells was determined using a microplate reader (Varioskan LUX, Thermo) at 570 nm to evaluate the cell viability.

### 2.6 Transwell assay

To assess migration and invasion, HGC27 and AGS cells were collected 24 h after transfection and/or CIRT treatment and resuspended in serum-free medium. For the migration assay, 2 × 10^4^ cells in 200 μL of serum-free medium were seeded into the upper chamber of a Transwell insert (8 μm pore size, 140,629, Thermo) without Matrigel coating. For the invasion assay, 2 × 10^4^ cells were seeded into Transwell inserts that had been pre-coated with Matrigel (356,234, Solarbio) for 1 h at 37 °C. In both the assays, 650 μL of complete medium supplemented with 10% FBS was added to the lower chamber of a 24-well plate to act as a chemoattractant. After 48 h of incubation, non-migrated or non-invaded cells remaining on the upper surface were gently removed with a cotton swab. Cells that had migrated or invaded the bottom side of the membrane were fixed with 4% paraformaldehyde (P0099, Beyotime) for 30 min and stained with 0.1% crystal violet (C0121, Beyotime) for another 30 min. Excess stains were washed away with distilled water. The stained cells were photographed and counted under an optical microscope (Leica DMi8 S, Germany).

### 2.7 Detection of iron level

The level of iron in HGC27 cells and AGS cells was examined using an iron assay detection kit (ab83366, Abcam, Cambridge, UK). In brief, after transfection, CIRT, or erastin treatment, cell homogenate was collected. Then, 50 μL of the cell homogenate was placed into each well, to which 5 μL of iron reducer and 5 μL of assay buffer were further added for 30 min incubation at 37 °C. After 100 μL, the iron probe was subsequently placed into each well for an hour incubation at 37 °C, and the OD value of each well was detected under a microplate reader at 593 nm wavelength for the iron levels calculation in line with the manufacturer’s manual.

### 2.8 Detection of reactive oxygen species level

The level of ROS in HGC27 cells and AGS cells was examined using a ROS assay kit with CM-H2DCFDA (S0035S, Beyotime) in line with the manufacturer’s manual. Briefly, after transfection, CIRT, or erastin treatment, HGC27 cells and AGS cells were incubated with CM-H2DCFDA for 30 min at 37 °C, and the CM-H2DCFDA was pre-diluted using FBS-free medium at a ratio of 1:1000. Then, the cells were washed with phosphate buffer solution (PBS; P1020, Solarbio). Finally, the ROS level was examined using a fluorescence microplate reader (SpectraMax Gemini EM, Molecular Devices, Shanghai, China).

### 2.9 Flow cytometry

The macrophage polarization was determined using flow cytometry. After the M0 macrophages were incubated with the culture supernatant of HGC27 cells and AGS cells after transfection, CIRT, or erastin treatment, the macrophages were collected and incubated with Allophycocyanin (APC)-conjugated anti-CD86 antibody (ab134385, Abcam) overnight at 4 °C, followed by incubating with PE-conjugated anti-CD206 antibody (ab318396, Abcam) overnight at 4 °C. Finally, the CD86^+^CD206^−^ cells were determined using a flow cytometer (Attune NxT, Thermo) to evaluate the macrophages’ polarization. Flow cytometry gate controls are shown in Supplementary Figure S2.

### 2.10 ELISA assay

The macrophage polarization from M0 to M1 was further evaluated by detecting the levels of markers in M1 macrophages (TNF-α, IL-6, and IL-1β). The human TNF-α ELISA kit (EK-H12145), human IL-6 ELISA kit (EK-H10352), and human IL-1β ELISA kit (EK-H10327) were obtained from EK Bioscience (Shanghai, China). Briefly, M0 macrophages were incubated with the culture supernatant of HGC27 or AGS cells after transfection, CIRT, or erastin treatment. After incubation, macrophage homogenates were collected, and 50 μL of each homogenate was added to the corresponding sample well. Subsequently, 100 μL of HRP-conjugated antibody specific to the target cytokine was added to each well and incubated for 60 min at 37 °C. Following incubation, the wells were washed five times with 350 μL of wash buffer, with each wash lasting 1 min. Next, 50 μL of substrate solution A and 50 μL of substrate solution B were added to each well and incubated in the dark at 37 °C for 15 min. The reaction was stopped by adding 50 μL of stop solution to each well. The optical density (OD) at 450 nm was measured using a microplate reader to quantify the levels of TNF-α, IL-6, and IL-1β in the macrophage samples.

### 2.11 Ethical statements and animal experiments

Eighteen male BALB/c nude mice (6 weeks old, 18–22 g) were obtained from Cavens Laboratory Animal Co., Ltd. (Changzhou, China) and housed under specific pathogen-free (SPF) conditions with a 12-h light/dark cycle and ad libitum access to food and water. All procedures were approved by the Ethics Committee of Gansu University of Chinese Medicine (Approval No. GCM202401025) and conducted in accordance with relevant national and institutional guidelines. Mice were randomly divided into three groups (*n* = 6 per group): the control group, the CIRT group, and the DHODH+CIRT group. All mice were subcutaneously injected into the right dorsal flank with 200 μL of PBS containing 3 × 10^5^ AGS cells. The control group received unmodified AGS cells, the CIRT group received AGS cells transfected with an NC plasmid, and the DHODH+CIRT group received AGS cells transfected with a DHODH overexpression plasmid. Tumor volume was measured every 3 days using the formula: tumor volume = 0.5 × width^2^ × length (30). When tumors reached approximately 100 mm^3^, mice in the CIRT and DHODH+CIRT groups were subjected to carbon ion irradiation using the IONTRIS raster-scanning system (Siemens, Munich, Germany) with a horizontal beamline at 333.82 MeV/u. Each tumor-bearing mouse was immobilized in a custom-made Lucite jig, and lead shielding was applied to protect non-tumor areas. A localized 4 Gy physical dose was delivered once daily for three consecutive days, targeting only the subcutaneous tumor through a shield window. Tumor volumes and body weights were monitored throughout the experiment. Mice were euthanized on day 30 post-tumor implantation (i.e., 3 days after the final irradiation), and tumors were excised for further volume, weight, gene, and protein expression analyses.

### 2.12 Western blot

The total proteins in the cells and mouse tumors were isolated using a protein extraction kit (KGB5303-100, KeyGEN, Jiangsu, China). Then, the total protein concentration was determined using a BCA protein assay kit (KGB2101-5000, KeyGEN). After the protein was incubated with SDS-PAGE loading buffer (KGC4501-1, KeyGEN) for 5 min at 100 °C, it was electrophoresed under SDS-PAGE gel (K4136, APExBIO) and further transferred to a Polyvinylidene Fluoride membrane (PVDF) membrane (KGC4805-5, KeyGEN), followed by incubation with 5% no-fat milk for 2 h. Then, the membrane was incubated with a relative primary antibody overnight at 4 °C and a secondary antibody for 2 h successively. After the membrane was covered with chemiluminescent substrate detection reagent (K1129, APExBIO), the protein signal was examined by Image Lab 3.0 (Bio-Rad, Hercules, California, USA). Antibody information in this assay was as follows: acyl-CoA synthetase long-chain family member 4 (ACSL4; 1:20000, ab155282, Abcam), glutathione peroxidase 4 (GPX4; 1:1000, #52455, CST, Boston, Massachusetts, USA), β-actin (1:3000, #4967, CST), and goat-anti-rabbit IgG (1:20000, ab6721, Abcam). Original images are shown in Supplementary Figure S3.

### 2.13 Quantitative real-time PCR (qRT-PCR) assay

The total RNA in cells and mouse tumors was isolated with the help of an RNA extraction reagent (K1621, APExBIO). Then, a UV spectrophotometer (NanoDrop; Thermo) was used to determine the total RNA concentration. Next, RNA was synthesized into cDNA through a reverse transcription kit (D7168S, Beyotime). Subsequently, the mixture of SYBR green reagent (D7260, Beyotime), cDNA, and primers of related genes was placed into a QuantStudio 6 System (Thermo) for the amplification reaction to determine the relative expression of related genes. The primers of related genes were as follows: DHODH forward primer: 5′-CCACGGGAGATGAGCGTTTC-3′, DHODH reversed primer: 5′-CAGGGAGGTGAAGCGAACA-3′, TNF-α forward primer: 5′-CCTCTCTCTAATCAGCCCTCTG-3′, TNF-α reversed primer: 5′-GAGGACCTGGGAGTAGATGAG-3′, IL-6 forward primer: 5′-CCTGAACCTTCCAAAGATGGC-3′, IL-6 reversed primer: 5′-TTCACCAGGCAAGTCTCCTCA-3′, IL-1β forward primer: 5′-ACTCACCTCTTCAGAACGAATTG-3′, IL-1β reversed primer: 5′-CCATCTTTGGAAGGTTCAGGTTG-3′, NOS2 forward primer: 5′-TCATCCGCTATGCTGGCTAC-3′, NOS2 reversed primer: 5′-CCCGAAACCACTCGTATTTGG-3′, β-actin forward primer: 5′-CACCATTGGCAATGAGCGGTTC-3′, and β-actin reversed primer: 5′-AGGTCTTTGCGGATGTCCACGT-3′. The relative mRNA expression levels were calculated using the 2^−ΔΔCt^ method. β-actin was used as the internal control.

### 2.14 Statistical analysis

All statistical analyses were performed using GraphPad Prism 9.5 (GraphPad Software, La Jolla, CA, USA). Data are presented as mean ± standard deviation of the mean (SD). Comparisons between two groups were conducted using an unpaired two-tailed Student’s *t*-test, while multiple group comparisons were analyzed using one-way or two-way ANOVA with Tukey’s *post-hoc* test, as appropriate. All *in vitro* experiments were performed with at least three independent biological replicates, and the *in vivo* experiments included six mice per group. A *p*-value of <0.05 was considered statistically significant.

## 3 Results

### 3.1 CIRT inhibited the viability, migration, invasion, and DHODH expression, while promoting the ferroptosis of gastric cancer cells

**Figure 1:**
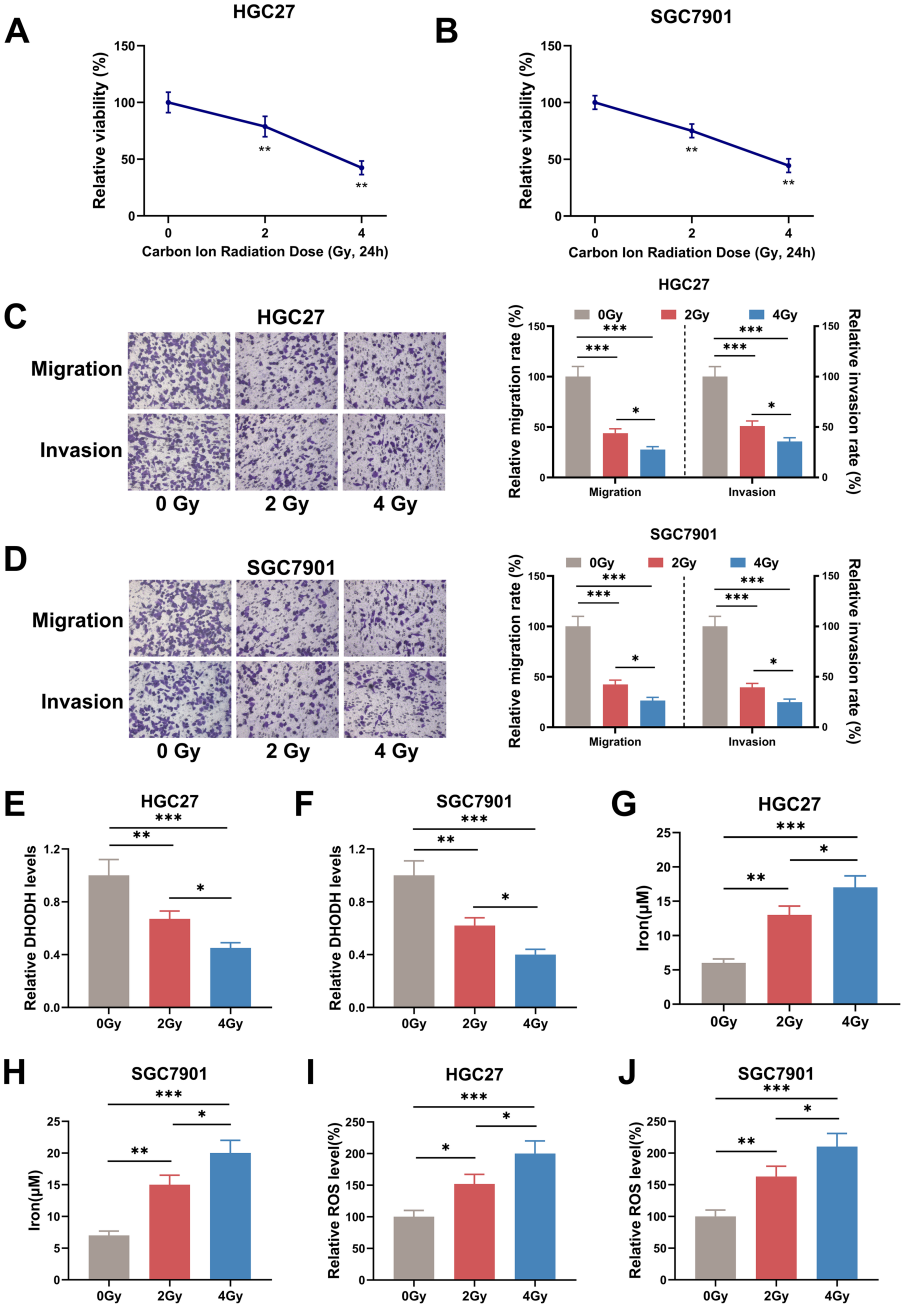
CIRT inhibited the viability, migration, invasion, and DHODH expression, while increasing the iron and ROS levels in gastric cancer cells. **(A–J)** After the HGC27 and AGS cells were treated with 0 Gy, 2 Gy, and 4 Gy of CIRT, the cell viability was determined using MTT assay **(A,B)**, the cell migration and invasion were detected by Transwell assay **(C,D)**, the expression of DHODH was quantified using qRT-PCR assay **(E,F)**, and the iron and ROS levels were examined by commercial reagent kits **(G–J)**. Bar charts **(C,D)** represent relative migration and invasion rates normalized to the 0 Gy group (100%). ^*^p < 0.05, ^**^p < 0. 01, ^***^p < 0.001.

**Figure 2:**
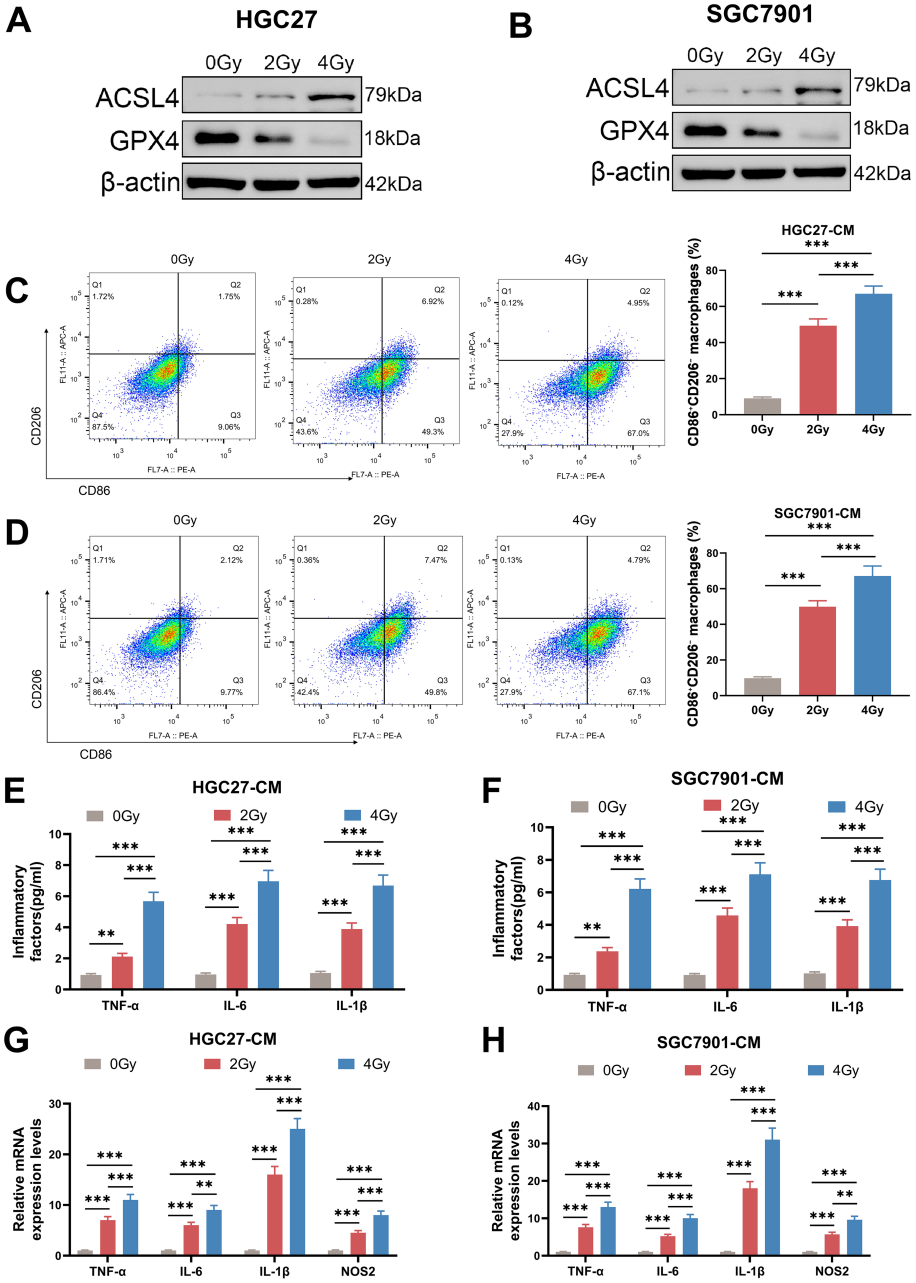
CIRT regulated the expression of ACSL2 and GPX4 of gastric cancer cells and induced the M0 macrophage polarization into M1 macrophages. **(A,B)** After the HGC27 and AGS cells were treated with 0 Gy, 2 Gy, and 4 Gy of CIRT, the expressions of ACSL2 and GPX4 were determined using Western blot. **(C–H)** After the M0 macrophages were treated with the culture supernatant of CIRT-treated HGC27 and AGS cells for 24 h, the markers of M1 macrophage polarization in macrophages were determined using flow cytometry **(C,D)**, the secretions of TNF-α, IL-6, and IL-1β in macrophages were detected using ELISA assays **(E,F)**, and the expressions of TNF-α, IL-6, IL-1β, and NOS2 in macrophages were quantified using qRT-PCR assay **(G,H)**. ^**^p < 0. 01, ^***^p < 0.001.

The cell viability of HGC27 cells and AGS cells after CIRT was first evaluated, as shown in Figures 1A,B, where both 2 Gy and 4 Gy doses of CIRT decreased the cell viability of HGC27 cells and AGS cells (*p* < 0.001). Meanwhile, as shown in Figures 1C,D 2 Gy and 4 Gy of CIRT reduced the ability of HGC27 and AGS cells to migrate and invade (*p* < 0.001). Besides, it was also found that CIRT decreased the expression of DHODH in both HGC27 and AGS cells (*p* < 0.01, Figures 1E,F). Subsequently, the iron and ROS levels were examined, and as shown in Figures 1G–J, both the iron level and ROS level in cells were increased by CIRT (*p* < 0.05). Besides, compared with 2 Gy CIRT, 4 Gy CIRT led to a more pronounced reduction in cell viability, migration, invasion, and DHODH protein expression, as well as a greater increase in intracellular iron and ROS levels (*p* < 0.05, Figures 1A–J). Furthermore, the expression of ferroptosis-related proteins was quantified, revealing that CIRT upregulated ACSL2 expression while downregulating GPX4 expression in both HGC27 and AGS cells (Figures 2A,B). These findings indicated that CIRT not only suppresses cell viability, migration, invasion, and DHODH expression but also promotes ferroptosis in gastric cancer cells.

### 3.2 CIRT induced M0 macrophage polarization into M1 macrophages

Macrophage polarization perturbs the tumor microenvironment and can trigger ferroptosis in cancer cells (32). Conversely, the induction of ferroptosis has been shown to promote the repolarization of M2 macrophages toward the antitumor M1 phenotype (33). We then evaluated whether CIRT could induce M0 macrophage polarization into M1 macrophages. After the M0 macrophages were treated with the culture supernatant of CIRT-treated HGC27 and AGS cells, markers of M1 macrophages were determined. As shown in Figures 2C,D, the culture supernatant of CIRT-treated HGC27 and AGS cells increased M1/M2 macrophages in a dose-dependent manner (*p* < 0.001). Besides, the culture supernatant of CIRT-treated HGC27 and AGS cells also upregulated the secretions or expressions of TNF-α, IL-6, IL-1β, and NOS2 in the macrophages in a dose-dependent manner (*p* < 0.01, Figures 2E–H), further demonstrating that CIRT had the ability to induce the M0 macrophage polarization into M1 macrophages in gastric cancer.

### 3.3 DHODH overexpression reversed the role of CIRT in inducing the ferroptosis of gastric cancer cells and inducing M1 macrophage polarization

**Figure 3:**
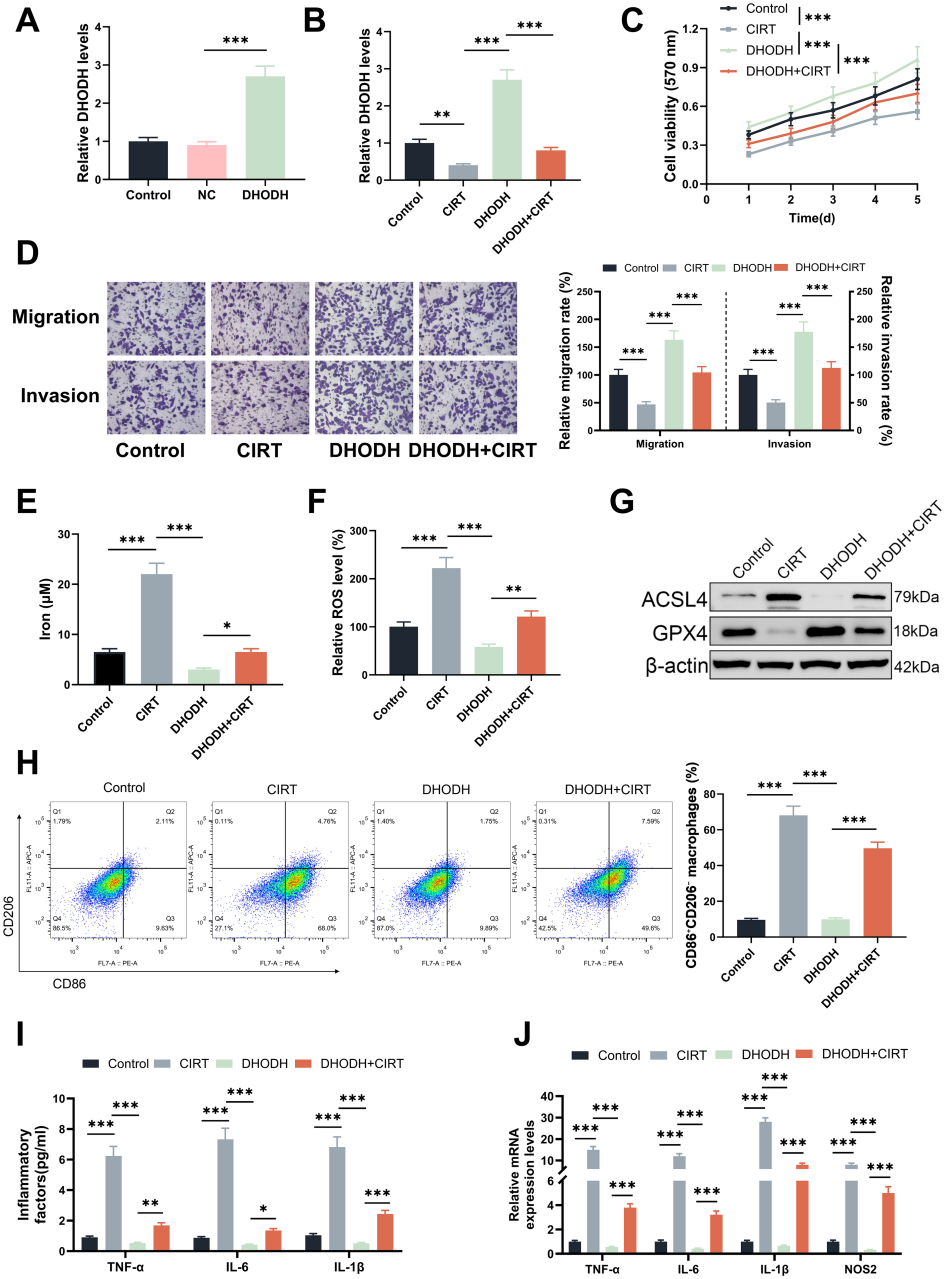
DHODH overexpression reversed the role of CIRT in inducing the ferroptosis of gastric cancer cells and in inducing M1 macrophage polarization. **(A)** The transfection efficiency of DHODH overexpression plasmids in AGS cells was determined using qRT-PCR assay (^***^p < 0.001 vs. NC). **(B–H)** After AGS cells were transfected with DHODH overexpression plasmids and treated with 4 Gy CIRT, the DHODH expression was quantified using qRT-PCR assay **(B)**, the viability was determined using MTT assay **(C)**, the migration and invasion were examined using Transwell assay **(D)**, the iron and ROS levels were detected using commercial reagent kits **(E,F)**, and the expressions of ACSL2 and GPX4 were determined using Western blot **(G)**. **(I–J)** After the M0 macrophages were treated with the culture supernatant of AGS cells, which were overexpressed DHODH before CIRT treatment, the markers of M1 macrophage polarization were determined using flow cytometry **(H)**, the secretions of TNF-α, IL-6, and IL-1β in macrophages were detected using ELISA assays **(I)**, and the expressions of TNF-α, IL-6, IL-1β, and NOS2 in macrophages were quantified using qRT-PCR assay **(J)**. Bar charts **(D)** represent relative migration and invasion rates normalized to the control group (100%). ^*^p < 0.05, ^**^p < 0.01, ^***^p < 0.001.

To determine whether the effects of CIRT on ferroptosis induction and M1 macrophage polarization in gastric cancer were mediated via DHODH, AGS cells were transfected with DHODH overexpression plasmids prior to 4 Gy CIRT treatment. As shown in Figures 3A,B, DHODH was downregulated by CIRT and upregulated by DHODH overexpression plasmids (*p* < 0.01), while the role of CIRT in DHODH expression was offset by DHODH overexpression (*p* < 0.001). CIRT significantly inhibited the viability (Figure 3C), migration (Figure 3D), and invasion (Figure 3D) of AGS cells, whereas DHODH overexpression markedly enhanced these malignant behaviors (*p* < 0.001). Moreover, the inhibitory effects of CIRT on these cellular functions were reversed by DHODH overexpression (*p* < 0.001). In addition, CIRT treatment significantly increased intracellular iron levels (Figure 3E), ROS levels (Figure 3F), and ACSL4 expression (Figure 3G), all of which were attenuated by DHODH overexpression (*p* < 0.001). Conversely, GPX4 expression was downregulated by CIRT and upregulated by DHODH overexpression. Notably, the CIRT-induced alterations in iron and ROS levels, as well as in ACSL4 and GPX4 expression, were all significantly mitigated by DHODH overexpression (*p* < 0.05, Figure 3G).

Meanwhile, the M0 macrophages were also treated with the culture supernatant of AGS cells, which overexpressed DHODH before CIRT treatment. As shown in Figure 3H, M1/M2 macrophages were increased by the culture supernatant of CIRT-treated AGS cells (*p* < 0.001) and decreased by the culture supernatant of DHODH overexpressed AGS cells (*p* < 0.001), and the role of CIRT in M1/M2 macrophages was offset by DHODH overexpression (*p* < 0.001). The levels of TNF-α, IL-6, IL-1β, and NOS2 in the macrophages were also upregulated by the culture supernatant of CIRT-treated AGS cells (*p* < 0.001, Figures 3I,J) and downregulated by the culture supernatant of DHODH overexpressed AGS cells (*p* < 0.001, Figures 3I,J). Besides, the role of CIRT in the levels of TNF-α, IL-6, IL-1β, and NOS2 in macrophages was reversed by DHODH overexpression (*p* < 0.001).

The above evidence confirmed that CIRT promoted the ferroptosis of gastric cancer cells and induced M1 macrophage polarization by downregulating DHODH.

### 3.4 Erastin reversed the role of DHODH overexpression in inhibiting the ferroptosis of gastric cancer cells and in inhibiting M1 macrophage polarization

**Figure 4:**
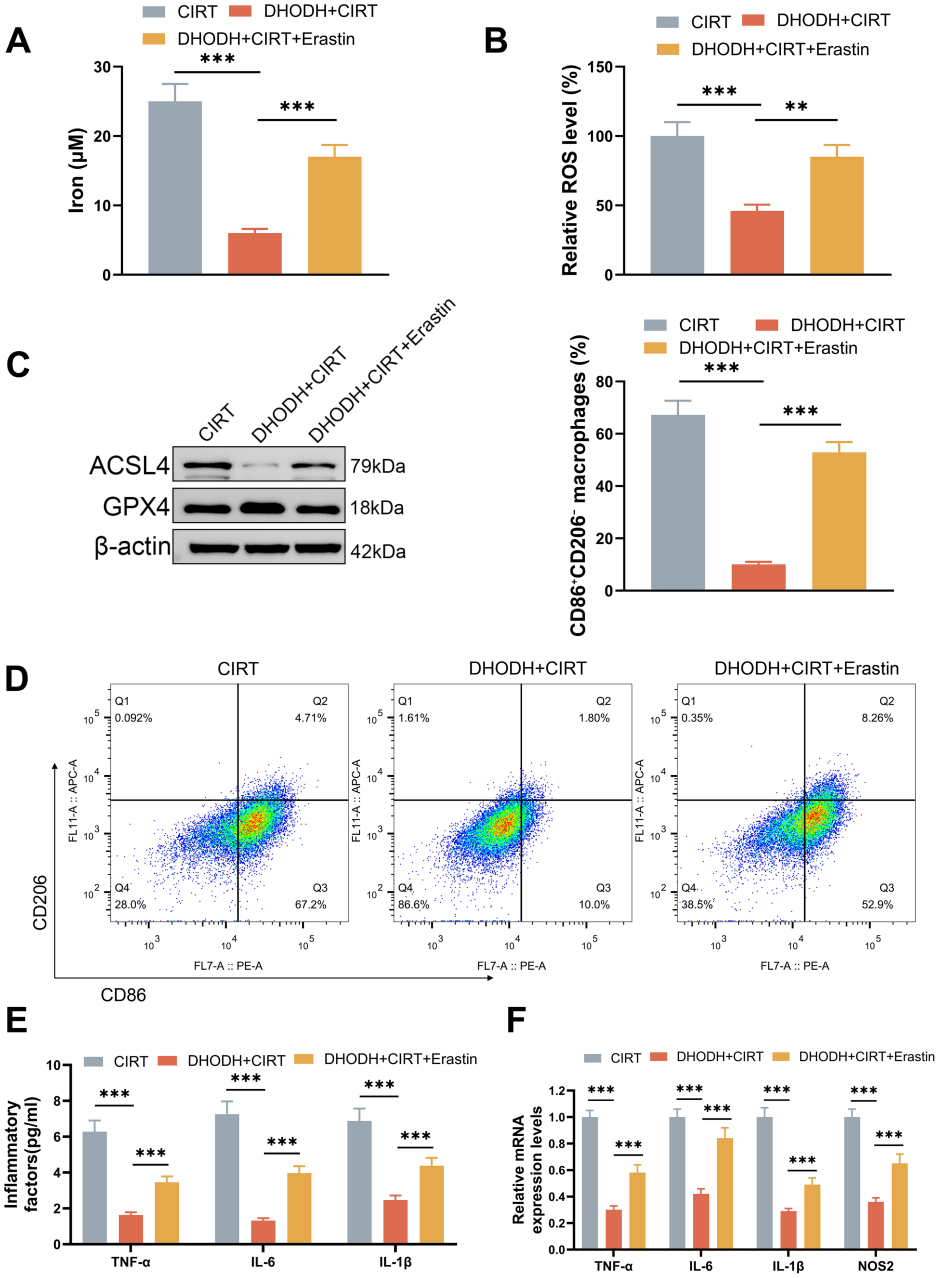
Erastin reversed the role of DHODH overexpression in inhibiting the ferroptosis of gastric cancer cells and in inhibiting M1 macrophage polarization. **(A–C)** After AGS cells were treated with 40 μM ferroptosis inducer erastin for 24 h after transfection and CIRT treatment, the iron and ROS levels were detected using commercial reagent kits (A, B), and the expressions of ACSL2 and GPX4 were determined using Western blot **(C)**. **(D–F)** After the M0 macrophages were treated with the culture supernatant of AGS cells, which were treated with erastin and CIRT after transfection, the markers of M1 macrophage polarization were determined using flow cytometry **(D)**, the secretions of TNF-α, IL-6, and IL-1β in macrophages were detected using ELISA assays **(E)**, and the expressions of TNF-α, IL-6, IL-1β, and NOS2 in macrophages were quantified using qRT-PCR assay **(F)**. ^*^p < 0.05, ^**^p < 0.01, ^***^p < 0.001.

Furthermore, 40 μM ferroptosis inducer erastin was used to treat the AGS cells for 24 h after transfection and CIRT treatment. As shown in Figures 4A–C, the iron level (Figure 4A), ROS level (Figure 4B), and ACSL4 expression (Figure 4C) in AGS were reduced by DHODH overexpression (*p* < 0.001), and the GPX4 expression (Figure 4C) was promoted by DHODH overexpression (*p* < 0.001); besides, the roles of DHODH overexpression in iron level, ROS level, and expressions of ACSL4 and GPX4 were offset by erastin (*p* < 0.01). Meanwhile, M0 macrophages were also treated with the culture supernatant of AGS cells, which were treated with erastin after transfection and CIRT treatment. As shown in Figure 4D, M1/M2 macrophages were decreased by the culture supernatant of DHODH-overexpressed AGS cells (*p* < 0.001), and the role of DHODH overexpression in M1/M2 macrophages was offset by erastin (*p* < 0.01). The levels of TNF-α, IL-6, IL-1β, and NOS2 in macrophages were significantly elevated following exposure to the culture supernatant of AGS cells overexpressing DHODH (*p* < 0.001, Figures 4E,F). However, this effect was reversed by treatment with erastin (*p* < 0.05, Figures 4E,F). These findings further support that DHODH inhibits M1 macrophage polarization by inhibiting ferroptosis in gastric cancer cells.

### 3.5 CIRT inhibited the growth of gastric cancer cells *in vivo* by downregulating DHODH

**Figure 5:**
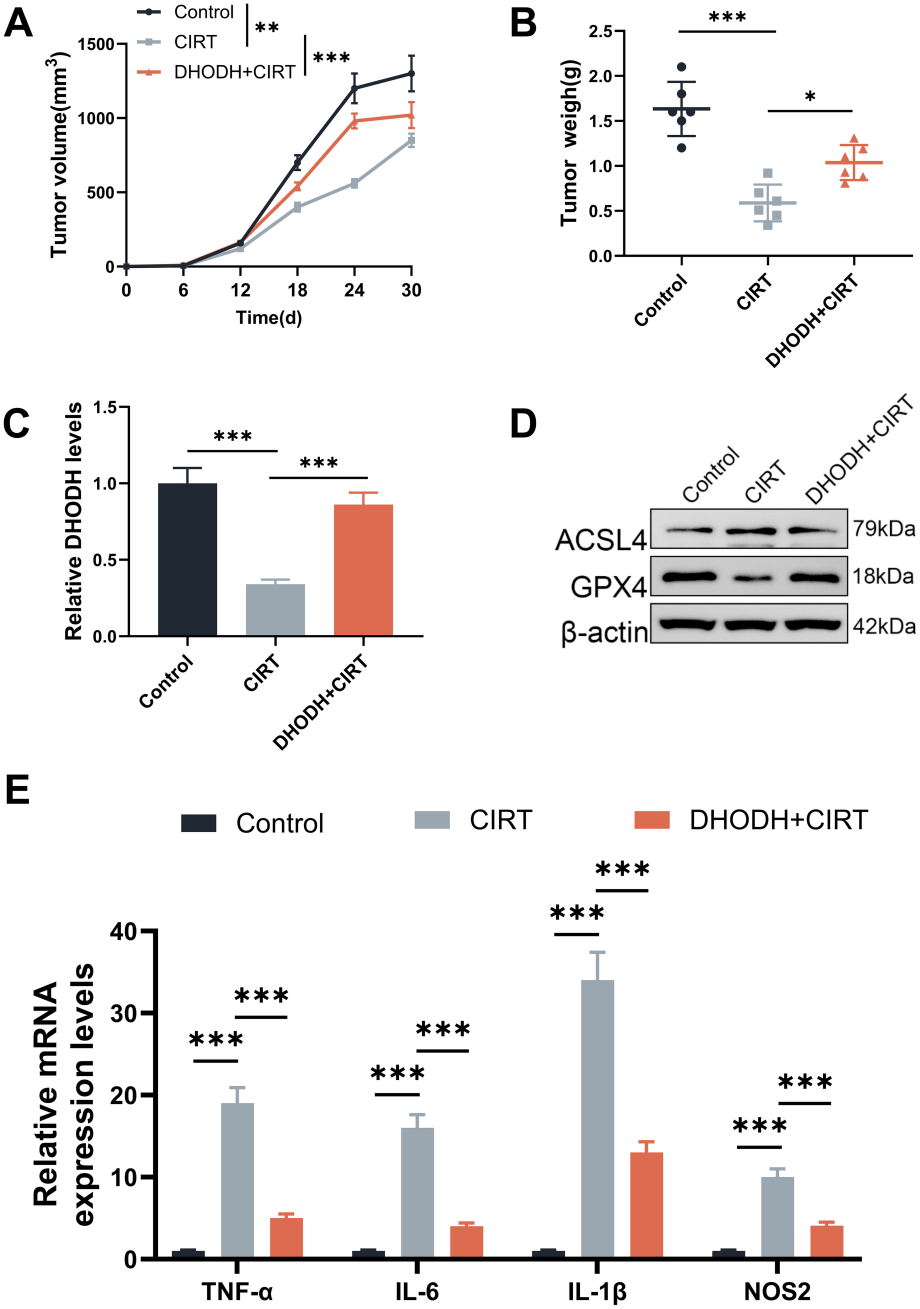
CIRT inhibited the growth of gastric cancer cells *in vivo* by downregulating DHODH. **(A–B)** After the xenograft tumor model was established, the tumor volume **(A)** and weight **(B)** were documented. **(C)** The expression of DHODH in tumor tissues was determined using qRT-PCR assay. **(D)** The expressions of ACSL2 and GPX4 in tumor tissues were quantified using Western blot. **(E)** The expressions of TNF-α, IL-6, IL-1β, and NOS2 in tumor tissues were examined by qRT-PCR assay. ^*^p < 0.05, ^**^p < 0.01, ^***^p < 0.001.

To further verify the above findings, animal experiments were performed. As shown in Figures 5A–B, CIRT treatment decreased the tumor volume and weight as compared to the control group (*p* < 0.01), while DHODH overexpression increased the tumor volume and weight in comparison to the CIRT group (*p* < 0.05). The expression of DHODH in tumor tissues was downregulated by CIRT (*p* < 0.001, Figure 5C), which was reversed by DHODH overexpression (*p* < 0.001, Figure 5C). Meanwhile, the expression of ferroptosis-related proteins in tumor tissues was also evaluated, as shown in Figure 5D, and CIRT upregulated ACSL4 and downregulated GPX4, which was further abolished by DHODH overexpression. Furthermore, the expression levels of TNF-α, IL-6, IL-1β, and NOS2 in tumor tissues were enhanced following CIRT treatment compared to the control group (*p* < 0.001, Figure 5E). However, these CIRT-induced elevations were markedly reduced by DHODH overexpression (*p* < 0.001, Figure 5E). These *in vivo* results further proved that CIRT induced ferroptosis and M1 macrophage polarization to inhibit the development of gastric cancer by downregulating DHODH expression.

## 4 Discussion

The present study found that CIRT decreased the viability, migration, and invasion capabilities of gastric cancer cells, suppressed DHODH expression and ferroptosis resistance, and promoted M1 macrophage polarization. Furthermore, these effects of CIRT on gastric cancer cells and macrophage polarization were mediated through the downregulation of DHODH. This study demonstrated that CIRT promotes ferroptosis and induces M1 macrophage polarization in gastric cancer cells, potentially through the downregulation of DHODH. These findings provide mechanistic insight into the anti-tumor effects of CIRT and suggest a potential role for DHODH as a therapeutic target in gastric cancer. CIRT is a kind of particle radiotherapy that uses a carbon ion beam to precisely target and destroy tumor cells (34). The main potential advantage of CIRT is that carbon ions accumulate more energy in smaller-volume tissues, causing greater damage to tumor cells. However, carbon ions have a characteristic dose distribution and are able to deliver high doses of radiation to tumors while minimizing damage to healthy tissue (35). A series of research has also proven the inhibitory effect of CIRT in cancers such as pancreatic cancer, prostate cancer, and lung cancer (36–38). In gastric cancer, for the first time, we discovered that CIRT weakened the viability and ability of gastric cancer cells to migrate and invade, and its in-depth regulatory mechanism needs further exploration.

As an enzyme essential for the *de novo* synthesis of pyrimidine, DHODH plays an important role in the pathogenesis of various diseases, such as cancer (39). Previous studies have reported that DHODH is upregulated in gastric cancer and that its overexpression is associated with chemoresistance and enhanced malignant behavior of gastric cancer cells (25–27). Similarly, the present study further confirmed that DHODH upregulation promotes the viability, migration, and invasion of gastric cancer cells. In addition, previous evidence has shown that DHODH inhibition enhances the radiosensitivity of cancer cells (28). In this research, it was found that CIRT reduced DHODH expression in gastric cancer cells, and the role of CIRT in reducing the viability and ability to migrate and invade of gastric cancer cells was offset by DHODH overexpression, demonstrating that CIRT inhibited the malignant characteristics by decreasing DHODH expression.

Furthermore, DHODH is an iron-containing flavin-dependent enzyme and is a crucial mediator of ferroptosis in different types of cancers (40). Ferroptosis is an iron-dependent regulation of cell death activated by extensive peroxidation of membrane phospholipids induced by oxidative stress (23). Downregulation of DHODH has been shown to induce ferroptosis and suppress the proliferation of cervical cancer cells (41), while DHODH inhibition-mediated ferroptosis induction has been reported to strengthen the radiosensitivity of nasopharyngeal carcinoma cells (28). Collectively, these findings suggest that DHODH inhibition-induced ferroptosis may represent a promising strategy for cancer treatment. Additionally, the sensitivity of liver cancer cells to high LET carbon-ion radiation has been associated with ferroptosis induction (42), indicating that the regulatory mechanism of CIRT might be associated with ferroptosis. GPX4, a central component of the ferroptosis defense systems, inhibits ferroptosis by reducing lipid hydroperoxides within membrane phospholipids (43). In contrast, ACSL4 is a key enzyme that catalyzes the biosynthesis of polyunsaturated fatty acid–containing phospholipids, thereby promoting ferroptosis (44). In the present study, CIRT significantly increased intracellular iron and ROS levels, upregulated ACSL4 expression, and downregulated GPX4 expression in gastric cancer cells. Notably, DHODH overexpression reversed these effects and abrogated the ferroptosis-inducing role of CIRT, indicating that CIRT promotes ferroptosis in gastric cancer cells through DHODH downregulation.

Recent studies have reported that macrophage polarization can disrupt the tumor microenvironment and induce ferroptosis in cancer cells (32, 45). Moreover, ferroptosis induction has been shown to facilitate the repolarization of M2 macrophages into the pro-inflammatory, antitumor M1 phenotype (33, 46). In this research, CM from CIRT-treated gastric cancer cells promoted macrophage polarization toward the M1 phenotype. In contrast, medium from DHODH-overexpressing gastric cancer cells suppressed M1 polarization and abolished the pro-polarizing effect of CIRT. Notably, the ferroptosis inducer erastin restored M1 polarization despite DHODH overexpression, indicating that CIRT drives M1 macrophage polarization in gastric cancer by downregulating DHODH.

To further verify the roles of CIRT and DHODH in gastric cancer cell growth, an *in vivo* xenograft tumor model was established. CIRT treatment significantly reduced tumor volume, tumor weight, and DHODH expression, while also modulating the expression of ferroptosis-related markers and macrophage polarization-associated factors in tumor tissues. These effects were reversed by DHODH overexpression, thereby reinforcing the *in vitro* findings and confirming that CIRT suppresses gastric cancer progression through DHODH downregulation. However, there are some limitations in this study. One limitation of our study is the absence of a direct comparison with conventional photon irradiation, which prevents a definitive conclusion regarding the superiority of CIRT in inducing ferroptosis or modulating macrophage polarization. Although previous studies have suggested potential advantages of CIRT due to its high LET and enhanced RBE, future investigations incorporating head-to-head comparisons with photons are necessary to validate these mechanistic differences in gastric cancer. Another limitation of our study is the absence of an *in vivo* group with DHODH overexpression alone (without CIRT), which would help to clarify the independent contribution of DHODH to tumor progression and immune modulation. Future studies will include such a group to further delineate the mechanistic role of DHODH in the tumor microenvironment. Besides, although tumor burden and treatment response were evaluated over a 30-day period, no survival data were collected, and thus, Kaplan–Meier analysis was not feasible. Future studies with longer observation periods and survival endpoints will be necessary to validate the long-term therapeutic efficacy of CIRT *in vivo*.

## 5 Conclusion

In conclusion, our results suggest that CIRT may induce ferroptosis and modulate macrophage polarization through DHODH-associated pathways in gastric cancer cells. While these findings provide preliminary mechanistic insights, further clinical investigations are required to validate the therapeutic potential of targeting DHODH in combination with CIRT.

## Data Availability

The raw data supporting the conclusions of this article will be made available by the authors, without undue reservation.
