# Supplementary material for: Carbon-ion radiotherapy induces ferroptosis and M1 macrophage polarization to inhibit the development of gastric cancer by downregulating DHODH
Source: Front Med (Lausanne). 2025 Aug 22;12:1592116. doi: 10.3389/fmed.2025.1592116 (PMC12411497; doi:10.3389/fmed.2025.1592116)
Supplement: Supplementary file 1 [file Data_Sheet_1.DOCX]

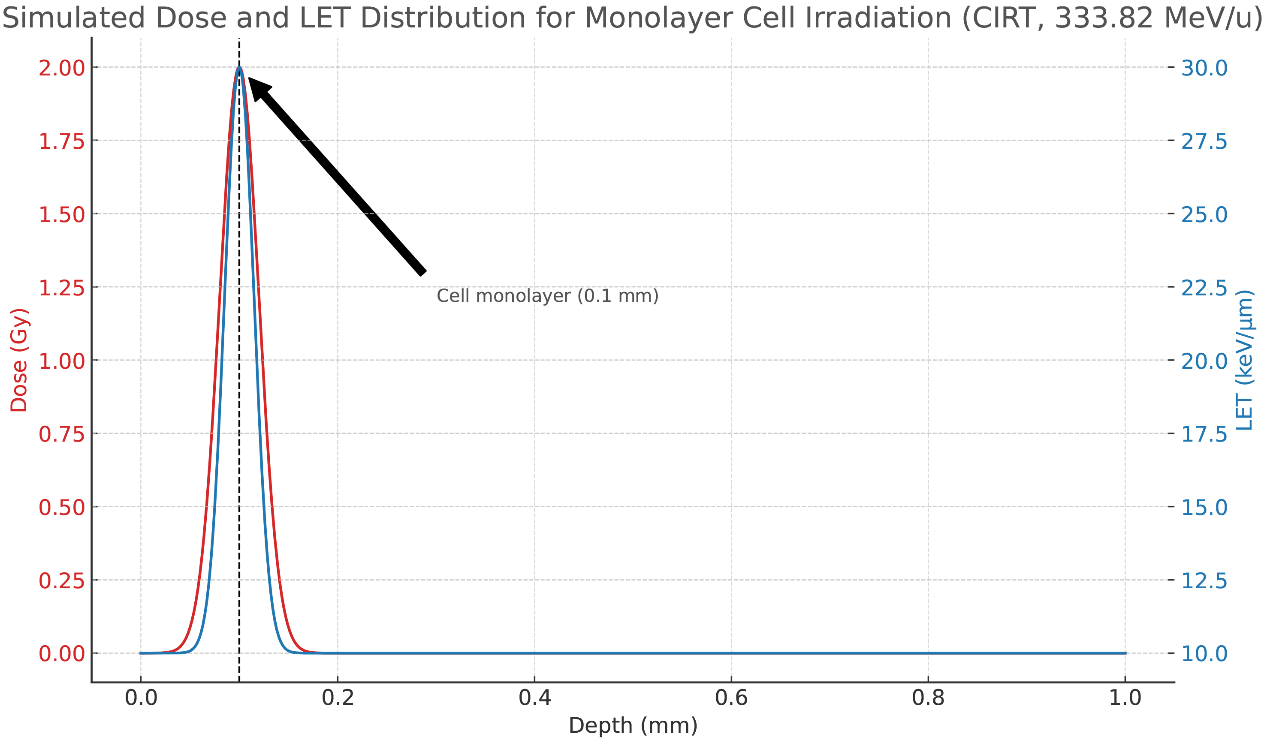


Figure S1. Simulated distribution of physical dose (red) and linear energy transfer (LET, blue) along the depth of a T25 flask for carbon-ion irradiation (333.82 MeV/u). The Bragg peak was adjusted to coincide with the monolayer of HGC27 and AGS cells adhered to the bottom surface (~0.1 mm). The LET at this location was approximately 29 keV/μm.


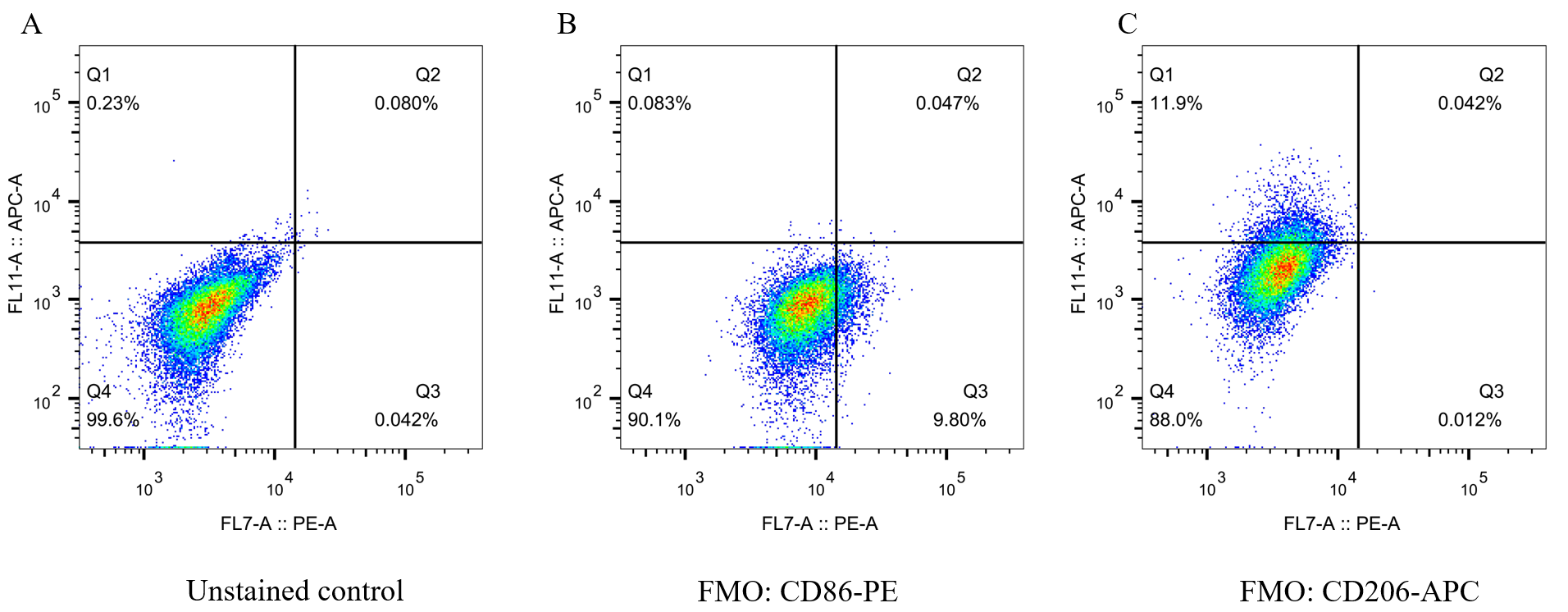


Figure S2. Flow cytometry gating controls. (A) Unstained control; (B) FMO-CD86; (C) FMO-CD206. These controls were used to define positive gating for CD86⁺CD206⁻ macrophages (M1-like phenotype).


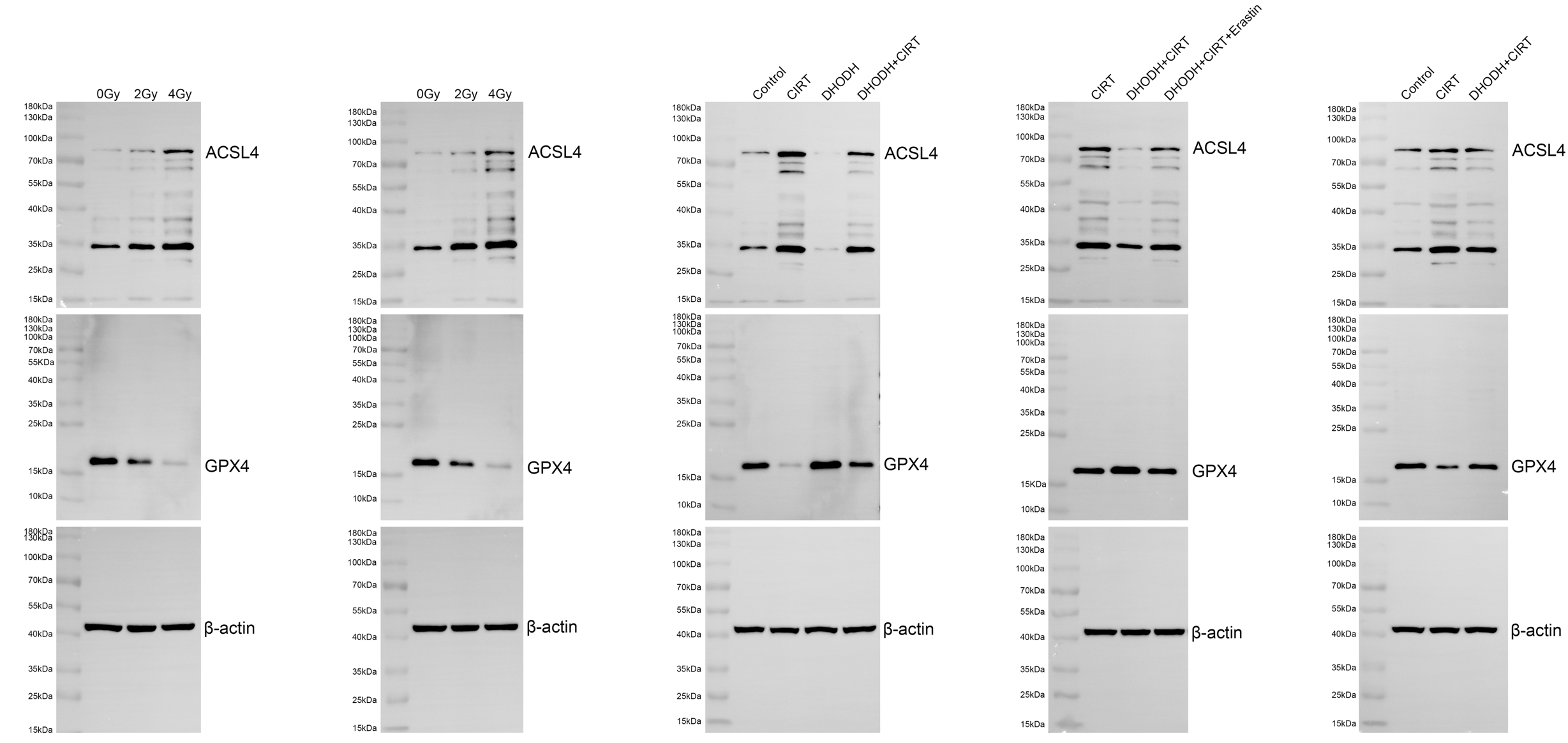


Figure S3. The original images of Western blot for the Figs. 2A, 2B, 3D, 4C and 5D.
